# Latent Classes of Envisioned Political Participation among Ethnic Minority and Majority Youth: The Role of Perceived Discrimination and Unfairness

**DOI:** 10.1007/s10964-026-02334-9

**Published:** 2026-03-19

**Authors:** Katrín Árnadóttir, Susanne Garritzmann, Nadja Wehl

**Affiliations:** 1 University of Konstanz, Konstanz, Germany; 2 Goethe University Frankfurt, Frankfurt, Germany

**Keywords:** Latent Class Analysis, political participation, ethnic minority and majority youth, perceived discrimination, perceived unfairness

## Abstract

Adolescence is a crucial period for political socialization in which attitudes toward politics and political engagement develop. Yet, research examining how youth envision their future political engagement in early adolescence is limited. Against the backdrop of persistent societal inequalities and discrimination across Europe, this study examined how ethnic minority and majority youth in Germany envisioned their future political engagement, and to what extent envisioned action or inaction coincided with perceptions of discrimination and unfairness. The sample consisted of 3033 youth (in the 7th grade, M_age_ = 12.59, SD_age_ = 0.67, 52% girls, 48% boys), 16.8% of which were classified as “ethnic minority” youth based on themselves or both of their parents not having been born in Germany. Using a person-centered approach (Latent Class Analysis), four latent classes of youth with qualitatively different patterns of envisioned political participation were identified: “voters” (47%), “unengaged” (26%), “engaged” (20%), and “undecided” (7%). In line with expectations, subsequent multinominal logical regression analysis showed that perceptions of systematic and/or group-based discrimination and unfairness robustly predicted membership in the engaged class over other classes, suggesting that such perceptions can promote envisioned political engagement among youth. While descriptive findings suggested that ethnic minority youth were more often unengaged and less often engaged than ethnic majority peers, multinominal logistic regression results showed that these differences were largely explained by other socio-economic factors.

## Introduction

Adolescence is a crucial formative period during which political attitudes and “political selves” crystalize – along with youths’ inclination to participate politically. Indeed, while political attitudes and inclinations to engage politically can develop and change throughout one’s life (Neundorf & Smets, 2017), some research suggests that political orientations and envisioned political engagement formed as early as at 11 or 12 years old can, for many youth, remain remarkably stable as they progress into adulthood (Jungkunz & Marx, 2025; Wray-Lake et al., 2014). Given the diverse forms of political participation available in modern democracies (Theocharis & van Deth, 2018), research among adults has increasingly highlighted the value of examining *patterns* of (envisioned or actual) political participation, that is, how and why different groups of people engage in or prefer certain political actions while avoiding others (Thomas et al., 2024). However, such work among youth remains more limited (but see e.g., Wray-Lake et al., 2014). Since the future political participation of youth is crucial for the long-term viability of democratic societies (Cammaerts et al., 2015), this highlights the relevance of investigating how youth envision their future political engagement at an early stage of adolescence, as their “political selves” begin to crystalize. Accordingly, the current study investigates patterns of envisioned political participation among youth, drawing on data from a large sample collected across secondary schools in Germany (provided by the project PerFair: “Students’ Perceptions of Inequality and Fairness”).

The current study adds to the literature in several ways. First, by adopting a person-centered approach and examining numerous forms of political participation (e.g., future voting, demonstrating, political consumerism, sharing political content online, working with a group towards social change), it identifies distinct groups of youth (i.e., “classes” in Latent Class Analysis) who differ in their envisioned political participation. Second, it focuses on youth with and without a migration background (referred to hereafter as “ethnic minority” and “ethnic majority” youth, respectively). Considering increasing migration-related diversity in many European countries, it is important to understand if and how *all* youth – including those with foreign origins – see themselves participating politically in the future. Not only does the political participation of ethnic minorities increase the credibility of democratic institutions (which are meant to serve the *whole* population, see e.g., Bäck & Soininen, 1998) – but it is also associated with greater social cohesion and reduced intergroup tensions within a society (Kende et al., 2024). Third, as part of externally validating the identified classes (e.g., Spurk et al., 2020), the current study tests whether several theoretically relevant variables differentiate between them (i.e., predict membership in one class over another). In particular, against the backdrop of persistent societal inequalities and well-documented discrimination faced by ethnic minorities in Europe and beyond (Carmo et al., 2018; Heath et al., 2008), the current study focuses on how perceptions of discrimination and unfairness – as potential drivers of (some forms of) political participation (Plummer et al., 2022; van Zomeren et al., 2008) – differentiate between the identified classes. Additionally, it explores whether ethnic minority (vs. majority) group membership differentiates the classes, and it accounts for various indicators related to youths’ socio-economic status, their gender, and their political interest.

### A Person-Centered Approach to Youths’ Political Participation

Many theories within psychology and the social sciences refer to groups or “types” of people who have certain characteristics or behaviors in common – and by extension, that differ from other groups along those dimensions. Thus, scholars frequently distinguish between “voters”, “activists”, and people that are “politically (un)engaged”, implying the existence of distinct groups of people whose political participation differs. Yet, political participation has, at least until recent years, most often been studied with so-called variable-centered approaches (e.g., regression analyses), that focus on relations between variables across individuals, based on an assumption of population homogeneity. In contrast, person-centered approaches (e.g., Latent Class Analysis) assume heterogeneity within a population, recognizing that variables can cluster together differently for distinct groups of people (see e.g., von Eye & Bogat, 2006). As such, person-centered approaches can identify more complex patterns or latent (sub)groups within a sample (“classes” in Latent Class Analysis, the method used in the current study), which would otherwise go undetected (e.g., Árnadóttir & Meeusen, 2024).

Substantial research among adults shows that person-centered approaches are a useful tool for advancing current understanding of political participation, by capturing how *different groups* of people *combine* different political actions (or lack thereof) in distinct ways (see Thomas et al., 2024 for a review). Such research has for instance empirically distinguished between individuals (i) primarily interested in voting, (ii) those more globally involved in a variety of actions and (iii) those not politically engaged (e.g., Jeroense & Spierings, 2023; Johann et al., 2020). Relatedly, by moving beyond examining *levels* of political participation and distinguishing instead between people who are interested in or that engage in *different kinds* of political actions, person-centered approaches can provide valuable insights into *why* groups of people engage in some actions and not others. For instance, prior research has distinguished between two groups engaged in actions aimed at reducing inequality, but which differed in *what kind* of actions they engaged in: “givers” donated goods or money, while “activists” engaged in actions aimed at challenging systemic inequality. Subsequent analyses showed that the groups could be differentiated (that is, predicted) by distinct emotions regarding societal inequality: “Givers” felt compassion towards disadvantaged groups, while “activists” felt outrage about the status quo (Thomas & McGarty, 2018, see also e.g., Baysu & Phalet, 2017). Had the authors simply aggregated these actions into a scale capturing an overall propensity to act (and/or more generally proceeded with a variable-centered approach), such insights would have been missed.

However, research adopting person-centered approaches to investigate the (envisioned) political participation of youth is more limited. Existing research adopting such approaches among youth includes research in Germany as well as Greece, examining adolescents from about 15 years of age. By differentiating broadly between “interpersonal” action (e.g., intervening if someone is bullied) and “structural” action (an aggregate of six distinct actions conceptually similar to some of those in the current study), three groups of youth were identified in a German sample, which differed clearly in levels of intended action (Schwarzenthal et al., 2024). The research in Greece examined actual participation, and found that while all youth were somewhat (or very) interested in politics, most of the sample (over 90%) reported low levels of actual participation, while two small groups reported differential participation (Kehl et al., 2025). Despite offering valuable insights, a limitation of both studies is that they aggregated several distinct actions (e.g., demonstrating, donating money) into a single scale, which may fail to capture heterogeneity in how youth combine or avoid distinct actions. Indeed, by distinguishing between several actions, a study conducted among among 8th grade students in the United States identified four distinct groups that differed both in their levels and patterns of envisioned participation (Wray-Lake et al., 2014; see also Waeterloos et al., 2021). Two groups did not envision much political participation (but differed e.g., in whether they envisioned staying informed on social issues). In contrast, two distinct groups did envision engagement: One group envisioned high engagement across items (e.g., voting and working to address social issues in the future), while the other primarily envisioned voting. Importantly, the authors tracked these students for four years (8th to 12th grade) and showed that most youth (about 75%) did not change in their patterns of envisioned participation (i.e., remained consistently in the same “group”). This suggests that envisioned engagement in early adolescence may already reflect relatively stable orientations that youth carry with them as they approach adulthood. The current study builds on and extends this work by examining – and distinguishing between – an even broader range of political actions, identifying distinct classes of youth (in Latent Class Analysis) who qualitatively differ in their envisioned political participation, in a large school-based sample.

### Predicting Membership in Political Participation Classes Among Youth

#### Perceptions of Discrimination and Unfairness

Many societies in Europe and beyond are characterized by persistent societal inequalities and discrimination (Carmo et al., 2018; Heath et al., 2008). For instance, it is well-documented that ethnic minority groups experience discrimination across domains such as the housing and labor markets (Flage, 2018; Quillian et al., 2019). This extends also to ethnic minority youth, whose minority status can make them targets of unfair and discriminatory treatment (Árnadóttir et al., 2022; Baysu et al., 2025). Germany is no exception: some research suggests that seven out of ten children and youth with a migration background and/or dark skin are regularly confronted with racism and discriminatory treatment (Götz, 2021). During adolescence, youth develop the capacity to recognize and reflect on social inequalities and the systemic disadvantages and privileges linked to social identities (Hazelbaker et al., 2022; McLoyd, 2019), and may thus become aware that they themselves or others face systemic disadvantage and discrimination due to factors such as ethnic group membership (e.g., Wray-Lake et al., 2023) or socio-economic status (e.g., Sutton, 2009).

The current study adds to the literature by examining how three distinct perceptions of discrimination and unfairness relate to youth’s envisioned political participation. First, it examines whether youth see themselves as belonging to a group that is discriminated against or disadvantaged in (German) society, captured by way of self-identification with such a group. According to the social identity approach (Tajfel & Turner, 1986), people derive part of their self-worth from the social groups they identify with and are thus motivated to maintain positive group identities. As such, perceiving one’s ingroup as discriminated against and/or disadvantaged can motivate political action to demand societal change towards equality (Ellemers & Van Laar, 2010; van Zomeren et al., 2008). Extensive evidence documents how identification with such a group and perceptions that one‘s ingroup is discriminated against is positively associated with many forms of (envisioned or actual) political participation, both among adults (van Zomeren et al., 2008, see also e.g., Árnadóttir et al., 2025) and youth (Hope & Jagers, 2014; Tyler et al., 2020). Accordingly, prior work identifying groups that differ in their (envisioned or actual) political participation among youth in Germany and Greece found that the more youth perceived that they were subject to discrimination due to their ethnic origins, the higher their (envisioned) participation (Kehl et al., 2025; Schwarzenthal et al., 2024). Importantly, however, perceptions of discrimination need not promote the use of all forms of political participation equally. For instance, research utilizing multiple rounds of European Social Survey data found that identification with a discriminated group was associated with a higher likelihood of demonstrating and contacting politicians, but a lower likelihood of voting (Mattila & Papageorgiou, 2017; see Bilodeau, 2017 for similar findings). However, it is worth noting that other work suggests that perceived discrimination on the basis of group membership can mobilize protests and activism, without undermining voting behavior (Delbasso, 2025; Tardivo, 2025).

Secondly, the current study captures more general perceptions of personal (un)fair treatment in life for unspecified reasons. While it is plausible that such general perceptions of *un*fair treatment may predict higher (envisioned) political participation (e.g., Zeng et al., 2018), research suggests that unlike perceived unfair or discriminatory treatment based on group membership, such general perceptions are often unrelated to political participation (Jalalzai, 2011; Wood & John-Henderson, 2024). This is in line with a social identity approach to political participation whereby perceptions of (and feelings regarding) *systemic and/or group-based* discrimination and unfairness are a critical ingredient of political action (van Zomeren et al., 2008).

Lastly, the current study examines youth’s perceptions of societal unfairness more generally, focusing on perceptions related to wealth inequality. Importantly, rather than simply capturing whether youth perceive that wealth is unequally distributed, which some youth may see as fair (as they might believe the rich have earned their wealth or otherwise deserve it; Biedron et al., 2024), and thus unproblematic (Jetten et al., 2021), the current study captures the extent to which youth perceive that the rich have more money than they *should have* compared to the poor. In this way, it gauges the extent to which youth perceive the wealth distribution to be (un)fair. Here, it is worth highlighting that *perceptions* related to economic inequality, as captured in the current study, should not be confounded with actual (objective) levels of economic inequality, as these inform political participation in different ways (Jo & Choi, 2019; Loveless, 2024). As with perceptions of discrimination on the basis of group membership, research suggests that disapproval of economic inequality may be more likely to promote less institutionalized activities (e.g., protests) rather than more institutionalized actions (e.g., voting; Loveless, 2024).

#### Ethnic Minority/Majority Group Membership and Socio-Economic Status

Substantial research has compared the political participation of ethnic minority and majority group members, with much work documenting an alleged “gap” in political participation, whereby ethnic minorities are less politically engaged than their majority peers (Ortensi & Riniolo, 2020; Torney-Purta et al., 2007). Yet, evidence for such a gap is mixed, as other research finds no difference or that ethnic minority group members are *more* politically active than their majority peers (Barrett & Pachi, 2019, pp. 70–71; Eckstein et al., 2015; Spies et al., 2020). Several factors may jointly explain these mixed findings. For instance, it may have to do with the form of political participation considered, as researchers have highlighted the need to consider varied forms of political participation so as to not underestimate the political engagement of ethnic minorities (Earl et al., 2017; Stepick et al., 2008). Other research has highlighted the potential role of socio-economic status. Higher socio-economic status can facilitate political engagement, as individuals with higher education and/or income may have more political knowledge and more time to devote to political participation (Brady et al., 1995; Verba et al., 1995). Since many ethnic minority groups face well-documented disadvantage in education and in the labor market across Europe (even when highly educated; Heath et al., 2008; Hermansen et al., 2025), differences in socio-economic status can play a role in explaining why ethnic minority group members have sometimes been found to be less politically engaged. Indeed, research shows that failing to account for such factors can result in the underestimation of the political engagement of ethnic minority youth (e.g., Lopez & Marcelo, 2008; Riniolo & Ortensi, 2021). Accordingly, the current research accounts for several indicators that jointly approximate family-based resources or disadvantage, which the current study broadly refers to as socio-economic status. Specifically, parental education, whether youth attend “Gymnasium” (a school type that prepares youth for entering university), educational resources in youth’s home, indicators of family affluence, and student’s German language skills are accounted for. Thus, the study combines indicators of both “cultural” and “economic/material” capital (see e.g., Sieben & Lechner, 2019) that are dimensions of socio-economic status.

#### Political Interest

In a last step, political interest will be accounted for. Political interest strongly and reliably predicts political participation among youth and adults alike – over and above factors such as (immigrant) origin, socio-economic status, and perceived discrimination (e.g., Bilodeau, 2017; Keating & Melis, 2017), which may inform political interest in the first place (e.g., Riniolo & Ortensi, 2021; Kleer et al., 2025). Furthermore, political interest may relate to perceptions of (societal) discrimination and unfairness, as politically interested individuals may be more aware of unequal distributions of resources and power across society.

## The Current Study Hypotheses

Research among adults has clearly illustrated the value of examining *patterns* of (envisioned or actual) political participation, that is, how and why different groups of people engage in or prefer certain political actions while avoiding others. However, such work remains more limited among youth. Accordingly, the current study adds to existing research by examining patterns of envisioned political participation at an early stage of adolescence and examines how perceptions of discrimination and unfairness inform these. As person-centered methods are inductive and data-driven in nature, the researchers refrained from formulating concrete hypotheses. However, based on existing findings among both adults and youth, it is expected that at least three classes will be identified, consisting of youth that: (i) do not envision much political participation, (ii) primarily envision voting, and (iii) envision engaging in multiple/varied actions in the future, respectively.

As for the focal predictors of class membership, it is expected that both identification with a group that is disadvantaged/discriminated against in Germany and perceptions of unfair distribution of wealth will predict more political engagement, in particular membership in a class characterized by envisioned engagement in a range of actions (i.e., a globally engaged, “future activist” class). In contrast, general perceptions of unfair treatment are not expected to predict class membership. Furthermore, the current study explores whether belonging to an ethnic minority (vs. majority) group predicts class membership. In line with recommendations to recognize heterogeneity within categories of “migration background” and/or “ethnic minority and majority groups” (see Civitillo et al., 2025), the current study does do not start from an assumed difference based on such categories. Rather, participatory classes are identified in the full sample, followed by *exploring* if ethnic majority/minority group membership predicts membership in a particular participatory class. As for other variables that are accounted for in the analysis, it is expected that both political interest and the various indicators related to youth’s (and their parents’) socio-economic status will predict membership in more politically active classes. The current study will also account for gender, but without forming expectations as to how it may predict class membership.

## Method

### Participants and Procedure

To empirically investigate classes of political participation among youth and predictors of class membership, the current study utilizes data on ca. 12-year-old adolescents (7th graders, M_age_ = 12.59, SD_age_ = 0.67, 52% girls, 48% boys) in three German States in 2022–2023 provided by the PerFair project (“Students Perceptions of Inequality and Fairness”). Supplementary Online Materials (SOM), along with the data and analysis code used for this study can be accessed at https://osf.io/h9n8x/. Data collection was approved by the ethics committee of the host university of the project as well as by the educational ministries of the three German states. Schools were sampled from a comprehensive list of all schools within each of the states, but due to the voluntary nature of the study, many contacted schools did not participate in the study, resulting in a participation rate of almost 6% of contacted schools. In schools willing to participate, students needed parental consent to take part in the study. Within participating schools, 3119 students participated (across 200 classes in 78 schools), which amounts to an individual response rate of close to 70%. However, since 83 students did not respond to the items measuring envisioned political participation, the sample used in this study amounts to 3033 students. The Supplementary Online Materials (SOM) offers further information on the sampling procedure and data collection (Sect.  1), and on (handling of) missing values (Sect.  2).

### Measures

**Envisioned political participation** was assessed using 12 indicators, which were the basis for the latent classes. Youth read: “…*what opportunities for political participation can you imagine using in the future? In the future*,* can you imagine …”* followed by: *(a) voting in federal elections*,* (b) being a candidate in an election (e.g.*,* for mayor)*,* (c) sharing a political post on Instagram*,* TikTok or similar*,* (d) working for social change in a political group (e.g.*,* Fridays for Future)*,* (e) signing a petition*,* (f) buying something for political reasons (e.g.*,* FairTrade products)*,* (g) not buying something for political reasons (e.g.*,* boycotting a brand)*,* (h) working with a youth organization of a political party*,* (i) writing a letter or an e-mail to a politician*,* (j) writing a political article for a blog or a (school) article*,* k) taking part in a demonstration*,* l) wearing T-shirts*,* sweaters*,* stickers etc. with a political message*. Response options were (i) *yes*, (ii) *maybe*, or (iii) *no.* This measurement of envisioned or intended political participation is standard in research on political socialization (e.g., Persson, 2012) and similar measures have been used in e.g., the “International Civic and Citizenship Education Study (ICCS)”.

**Identification with a group that is disadvantaged and/or discriminated** in society was measured with the question “Would you say that you belong to a group that is discriminated against or disadvantaged in Germany?” (0 = *no*, 88.1%; 1 = *yes*, 11.9%).

**Perception of unfair distribution of wealth** was measured by first asking youth how much money they thought the richest people in Germany *actually* had compared to the poorest people (1 = *a little more money*, 5 = *a lot more money*). Next, they were asked how much money they thought the richest people in Germany *should have* compared to the poorest (1 = *a little more money*, 5 = *a lot more money*). From these two items, a difference score was calculated (range − 4 to + 4) with more positive scores indicating that youth perceived the rich to have more money *than they should have* (*M* = 1.33, *SD* = 1.29). This indirect measure of perceptions of unfair distribution of wealth follows the logic in justice research, which defines unfairness as (mis)match between a perceived just distribution and the perceived actual distribution (Jasso & Wegener, 1997).

**Perceived personal fair treatment** was measured with the item “I am treated fairly in life” (1 = *do not agree at all*, 4 = *strongly agree*, *M* = 2.83, *SD* = 0.77). Note that in contrast to the previous items on *un*fairness and discrimination, in this case higher values refer to higher perceived personal fair treatment.

**Ethnic minority or majority group membership** was defined by whether youth had an immigrant background, based on youth’s and their parent’s country of birth. Specifically, students were coded as ethnic minority students if they or both of their parents were born in a different country than Germany (*N* = 498, 16.8% of the sample; see “additional analysis” below for exploratory analyses involving (i) youth with one German parent and (ii) citizenship status).

**Gender** was accounted for (self-reported, 0 = *Girl*, 52%; 1 = *Boy*, 48%), as prior research frequently reveals gender differences in political participation (e.g., Zani & Barrett, 2012). Youth that preferred not to respond (2.1%) were excluded from analyses involving gender.

**Parental education** was assessed with information regarding whether parents had “Abitur”, a qualification required for access to university in Germany (based on parent information when available, otherwise using youth’s responses). However, as this measure had substantial missing values (18%; with youth in some latent classes reporting many more missing than others; see Sect.  2 in SOM for further information), models were tested both with and without parental education. For parsimony – and as findings largely replicated across these models – models without parental education are presented in the main text (for models with parental education, see Sect.  3 in SOM).

**School type** was captured by coding whether youth attended a *Gymnasium* – a school type leading to the “Abitur”, the qualification needed to enter university – as compared to other schools that do not explicitly prepare youth for university education (0 = *Gymnasium*, 42.3%; 1 = *Other school types*, 57.7%).

**Books in the home** were captured by asking youth to estimate how many books there were in their home *(*1 = *0–25*, 5 = *500 or more*). This measure is frequently used in research among youth, including in Germany – and has been found to predict higher (intended) political engagement (e.g., Schwarzenthal et al., 2022).

**Language skills** of students were captured as share of correct answers on language tests assessing German language skills and proficiency (coded on a scale from 0 to 1, *M* = 0.84, *SD* = 0.13).

**Family affluence** was captured with two items from the family affluance scale (Boyce et al., 2006), which is frequently used in research, including among youth in Germany (e.g., Richter & Leppin, 2007). Students were asked if they “went on vacation with their parents at least once a year” and if “they had their own room” (0 = *no*, 1 = *yes*). An index was created by averaging these two items (range: 0–1, *M* = 0.88, *SD* = 0.24).

**Political interest** was measured with the item “How interested are you in politics” (1 = *not at all*, 4 = *very much*, *M* = 2.56, *SD* = 0.97).

Although most items, particularly those referring to political engagement, were taken from established surveys among youth, such as the *International Civic and Citizenship Education Study*, the project team conducted extensive pretests to ensure a high quality of the responses. Firstly, cognitive pretests served to investigate whether youth understood core concepts as well as specific items the way they were intended by the project team; second, a pilot study was conducted with the adapted questionnaire to test the overall duration, concentration spell of youth, and implementation procedures of the survey before the main field phase.

### Analytic Strategy

All analyses were conducted using Mplus 8.11 (Muthén & Muthén, 2017). First, intraclass correlations (ICCs) were examined, which estimated the impact of classroom-level clustering on our focal measures capturing envisioned engagement. The ICCs indicated that the proportion of variance at the classroom-level was overall low (0.069 on average for the indicators of envisioned action). Thus, when considered together with the fact that the classroom-level is not directly relevant for the current study’s research questions, analyses were conducted without a multilevel approach, applying maximum likelihood estimation with robust standard errors (MLR), to account for non-normality in our measures (as in e.g., see Schwarzenthal et al. (2024).

Regarding the Latent Class Analysis (LCA) and subsequent explanatory model, a three-step approach was adopted. In the first step, a LCA of the 12 items capturing youth’s envisioned political participation was conducted. The appropriate number of classes was determined by estimating several models with increasing number of classes and comparing them based on several criteria of model fit (Bauer, 2022; Nylund et al., 2007). More specifically, the entropy was examined – an indicator of classification quality – with values of 0.8 and above indicating good classification quality (Clark & Muthén, 2009). Moreover, lower values on the Bayesian information criterion (BIC), the sample size-adjusted BIC (aBIC), and the Akaike information criterion (AIC) signal a better model. In addition, a significant Likelihood Ratio Test (LRT) indicates that the model with K–1 classes is preferred to the model with K classes. Importantly, though, the “best” final solution is one that balances model parsimony and fit, and that delivers substantively interpretable and meaningful classes (Bauer, 2022). Moreover, while small classes should be retained if conceptually meaningful and supported by fit statistics, very small classes are undesirable. Lastly, the robustness of the final model was verified by running the LCA with 500 starting values (as recommended in e.g., Geiser, 2012), ensuring that the best log-likelihood value could be replicated. In a second step, after determining the appropriate number of classes, a variable assigning youth to one of the classes based on their posterior class membership probabilities was created. In a third step, a multinomial logistic regression was estimated using this class membership indicator as the dependent variable, whereby three sequential models were assessed. Model 1 included ethnic minority/majority group membership, gender, school type, family affluence, books in the home and language skills. Model 2 additionally included perceptions of discrimination and unfairness. Lastly, Model 3 further included political interest. Perceptions were assessed in Model 2, separate from political interest, as they are known to predict political interest (cf. supra). Similarly, ethnic minority/majority group-membership and socio-economic factors were assessed ahead of perceptions, as the former can inform the latter (e.g., ethnic minority youth are more likely to identify with a disadvantaged group). Testing these three models sequentially makes it possible to first establish whether ethnic minority/majority group membership and socio-economic status predict membership in political participation classes in their own right (rather than over and above identification with a disadvantaged/discriminated group, for instance) – and thereafter whether perceptions of discrimination and unfairness do so (over and above Model 1). Model 3 then shows which of the variables in Models 1 and 2 predict class membership over and above political interest, which should be most proximally related to the identified classes.

## Results

### Identifying Latent Classes of Ethnic Minority and Majority Youths’ Envisioned Political Participation

#### Selecting the number of classes (full sample)

As Table 1 shows, all statistical indicators indicated that a four-class solution fit the data better than a three-class solution: It had lower AIC, BIC and aBIC values, significant LRT tests, and higher entropy. Moreover, a new, relatively small (*N* = 201, 7% of total sample), class emerged in the four-class solution, which had a clearly distinct response pattern compared to the other classes. Comparing a four-class to a five-class solution, some statistical indicators suggested that the five-class solution fit the data better than the four-class solution, whereas others did not. Moreover, inspecting the five-class solution revealed that the 5th class was created by sub-dividing the largest class into two classes with substantively similar response patterns. Taken together, it was determined that the four-class solution fit the data best. In a last step, the stability of this solution was verified by running it with twice as many random starts (1000, retaining 250). The solution remained stable.

**Table 1:** Model comparisons (500 random starts, 125 retained after 100 iterations)

# Classes	aBIC	BIC	AIC	LMR LRT	VMLR LRT	Entropy	Class sizes
2 classes	65168.32	65324.01	65029.16	*p* < .001	*p* < .001	0.84	1685, 1348
3 classes	63863.95	64099.08	63653.8	*p* < .001	*p* < .001	0.801	1494, 967, 572
4 classes	62763.31	63077.87	62482.15	*p* < .001	*p* < .001	0.836	1439, 776, 617, 201
5 classes	62301.09	62695.08	61948.94	*p* < .001	*p* < .001	0.802	869, 861, 607, 487, 209
6 classes	61996.37	62469.8	61573.22	*p* = .010	*p* = .010	0.778	730, 594, 578, 569, 357, 205

#### Examining the four-class solution

Table 2 below presents an overview of the response patterns of each of the four classes (i.e., % of youth within each class that responded “yes”, “maybe” and “no” to each of the envisioned action items). To facilitate the visual detection of the response patterns of each class, higher response rates are shown in darker shades. When describing the classes hereafter, the term “interested” is used when a majority of youth in a class indicate that they can envision engaging in a particular action (i.e., > 50% respond “yes”), and the term “open” when the majority of responses are divided between “yes” and “maybe”, with only a minority of youth indicating they do *not* see themselves engaging in an action.

**Table 2:** Responses by class

As Table 2 shows, youth in the largest class (47%, *N* = 1439) were generally interested in, or at least open to, voting in the future, but did not clearly envision themselves participating in other political actions (as is evidenced by the relatively low rate of “yes” responses). However, they were relatively more open to engaging in some actions (e.g., signing a petition), than others (e.g., writing a letter or an email to a politician). Hereafter, this class is referred to as the “voting” class.

Youth in the second largest class (26%, *N* = 776) were only somewhat open to voting in the future and clearly did *not* see themselves participating politically in other ways. Hereafter, this class as referred to as the “unengaged” class.

Youth in the third largest class (20%, *N* = 617), in contrast, showed clear interest not only in voting in the future, but also in multiple other actions. Indeed, youth in this class can be characterized as interested in or at least open to all the listed political actions, even the most “demanding” actions, such as standing as a candidate in an election or working for a youth organization of a political party – which youth in all other classes were clearly *not* open for. Hereafter, this class is referred to as the “engaged” class.

The fourth and smallest class (7%, *N* = 201) consisted of youth who appeared to be undecided as to whether they wished to engage in political action in the future (responding “maybe” to all (or most) items). Hereafter, this class is referred to as the “undecided” class.

### Predicting Class Membership

#### Initial analysis / Descriptives

Table 3 shows the bivariate correlations between the external predictor variables. Belonging to an ethnic minority group is associated with lower resources and skills (e.g., lower language skills, less family affluence, fewer books in the home), and with a higher likelihood of identifying as a member of a disadvantaged/discriminated group, but with a lower perceived unfair distribution of wealth. The three indicators of discrimination and unfairness are only modestly correlated, indicating that these capture distinct perceptions (for means and standard deviations by class, see Sect.  4 in SOM).

**Table 3:** Correlations Between the External Predictors of Class Membership

	1		2		3		4		5		6		7		8		9	
1.Ethnic minority/majority group membership (ref: majority group)																		
2. Gender (ref: girls)																		
3. School type (ref: Gymnasium)	0.11***		0.05**															
4. Language skills	− 0.26***		0.16***		− 0.41***													
5. Family affluence	− 0.26***		0.05*		− 0.18***		0.16***											
6. Books in the home	− 0.29***		− 0.04*		− 0.39***		0.28***		0.19***									
7. Identification with a disadvantaged/discriminated group (ref: *Not* identifying)	− 0.18***		− 0.06***		0.08***		− 0.11***		− 0.16***		− 0.11***							
8. Perceived personal fair treatment	− 0.08***		0.07***		− 0.19***		0.10***		0.14**		0.13**		− 0.22***					
9. Perceived unfair distribution of wealth	− 0.17***		− 0.05**		− 0.16***		0.22***		0.04*		0.17***		− 0.04*		0.03			
10. Political interest	0.01		0.02		0.14***		0.10***		0.03		0.14***		0.07***		0.03		0.04*	

Notes.*p < .05, **p < .01, ***p < .001. Ref = reference

#### Multinominal Logistic Regression Analysis

Next, multinomial logistic regression models predicting class membership were estimated. As is outlined in the analytic strategy, three sequential models were assessed. For parsimony, results from Models 2 and 3 are presented in the main text, as shown in Tables 4 and 5 (see Sects.  5 and 3 in SOM for full Model 1 with and without parental education). In the model displayed in Table 4, the largest “voting” class was treated as a reference category. Table 5 presents the same model but with the “engaged” class as a reference category, enabling a direct comparison of this class with the “unengaged” and “undecided” classes. Comparisons between the “unengaged” and “undecided” classes are summarized in the text (see Sect.  6 in SOM for a table comparing these classes).

**Table 4:** Multinomial Logistic Regression Results (Models 2 and 3) with the “Voter” Class as the Reference Class

Predictors / Class	Engaged class (*N* = 570)										Unengaged class (*N* = 674)												Undecided class (*N* = 167)												
	Model 2				Model 3					Model 2							Model 3						Model 2							Model 3					
	Coeff (*SE)*		*p*	OR	Coeff (*SE)*		*p*	OR		Coeff (*SE)*			*p*	OR			Coeff (*SE)*			*p*	OR			Coeff (*SE)*		*p*	OR			Coeff (*SE)*			*p*	OR	
Ethnic minority/ majority group membership (ref: majority group)	0.07 *(0.18)*		0.680	1.076	-0.01 *(0.19)*		0.971		0.993	0.32 *(0.14)*			0.026			1.373	0.40 *(0.15)*			0.006		1.497	0.47 *(0.24)*			0.047			1.604	0.47 *(0.24)*			0.048		1.601
Gender (ref: girls)	-0.56 *(0.11)*		< 0.001	0.570	-0.77 *(0.12)*		< 0.001		0.461	0.64 *(0.10)*			< 0.001			1.890	0.72 *(0.10)*			< 0.001		2.062	0.12 *(0.17)*			0.477			1.128	0.12 *(0.17)*			0.487		1.127
School type (ref: Gymnasium)	-0.47 *(0.12)*		< 0.001	0.623	-0.45 *(0.12)*		< 0.001		0.636	0.49 *(0.12)*			< 0.001			1.628	0.48 *(0.12)*			< 0.001		1.611	0.38 *(0.20)*			0.057			1.466	0.39 *(0.20)*			0.053		1.476
Language skills	0.48 *(0.65)*		0.458	1.619	0.29 *(0.64)*		0.657		1.331	-0.51 *(0.47)*			0.278			0.600	-0.13 *(0.50)*			0.796		0.878	− 0.2.75 *(0.63)*			< 0.001			0.064	-2.74 *(0.63)*			< 0.001		0.064
Family affluence	0.39 *(0.25)*		0.121	1.472	0.36 *(0.26)*		0.156		1.436	-0.21 *(0.21)*			0.322			0.812	-0.21 *(0.21)*			0.327		0.810	0.26 *(0.40)*			0.513			1.298	0.27 *(0.40)*			0.505		1.307
Books in the home	0.23 *(0.04)*		< 0.001	1.259	0.21 *(0.04)*		< 0.001		1.235	-0.16 *(0.04)*			< 0.001			0.851	-0.13 *(0.04)*			< 0.001		0.877	0.11 *(0.06)*			0.076			1.118	0.11 *(0.06)*			0.078		1.120
Identification with a disadvantaged/ discriminated group ref: *not* identifying)	0.48 *(0.17)*		0.004	1.618	0.33 *(0.17)*		0.057		1.386	-0.35 *(0.17)*			0.038			0.704	-0.25 *(0.17)*			0.155		0.780	-0.53 *(0.31)*			0.088			0.586	-0.55 *(0.32)*			0.084		0.578
Perceived personal fair treatment	-0.01 *(0.07)*		0.939	0.994	− 0.01 *(0.07)*		0.903		0.991	-0.02 *(0.07)*			0.793			0.983	-0.01 *(0.07)*			0.945		0.995	-0.02 *(0.11)*			0.850			0.979	-0.02 *(0.11)*			0.875		0.982
Perceived unjust distribution of wealth	0.18 *(0.04)*		< 0.001	1.199	0.19 *(0.05)*		< 0.001		1.212	-0.05 *(0.04)*			0.190			0.949	-0.06 *(0.04)*			0.141		0.940	− 0.06 *(0.07)*			0.374			0.943	-0.06 *(0.07)*			0.363		0.941
Political interest					0.63 *(0.06)*		< 0.001		1.876								-0.62 *(0.06)*			< 0.001		0.540								0.01 *(0.09)*			0.932		1.008

Notes. The voter class is the reference category. Coeff = Logit coefficient, SE = Standard Error, OR = Odds Ratio, Ref = reference group. Voter class *N =* 1298

**Table 5:** Multinomial Logistic Regression Results (Models 2 and 3) with the “Engaged Class” as the Reference Class

Predictors / Class	Unengaged class (*N* = 674)										Undecided class (*N* = 167)									
	Model 2					Model 3					Model 2					Model 3				
	Coeff (*SE)*			*p*	OR	Coeff (*SE)*			*p*	OR	Coeff (*SE)*			*p*	OR	Coeff (*SE)*			*p*	OR
Ethnic minority / majority group membership (ref: majority group)	0.24 *(0.20)*			0.229	1.274	0.41 *(0.22)*			0.060	1.507	0.40 *(0.27)*			0.143	1.490	0.48 *(0.24)*			0.091	1.612
Gender (ref: girls)	1.20 *(0.130)*			< 0.001	3.316	1.50 *(0.14)*			< 0.001	4.470	0.68 *(0.19)*			< 0.001	1.980	0.89 *(0.19)*			< 0.001	2.443
School type (ref: Gymnasium)	0.96 *(0.15)*			< 0.001	2.615	0.93 *(0.15)*			< 0.001	2.535	0.86 *(0.22)*			< 0.001	2.355	0.84 *(0.22)*			< 0.001	2.323
Language skills	-0.99 *(0.72)*			0.167	0.371	-0.42 *(0.76)*			0.582	0.660	-3.23 *(0.82)*			< 0.001	0.039	-3.03 *(0.80)*			< 0.001	0.048
Family affluence	-0.59 *(0.28)*			0.035	0.552	-0.57 *(0.29)*			0.052	0.564	-0.13 *(0.43)*			0.771	0.882	-0.09 *(0.43)*			0.829	0.910
Books in the home	-0.39 *(0.05)*			< 0.001	0.676	-0.34 *(0.05)*			< 0.001	0.710	-0.12 *(0.07)*			0.083	0.888	-0.10 *(0.07)*			0.161	0.907
Identification with a disadvantaged/ discriminated group Ref: *Not* identifying)	-0.83 *(0.21)*			< 0.001	0.435	-0.58 *(0.22)*			0.008	0.563	-1.02 *(0.33)*			0.002	0.362	-0.88 *(0.34)*			0.009	0.417
Perceived personal fair treatment	-0.01*(0.09)*			0.889	0.988	0.00 *(0.09)*			0.964	1.004	-0.02 *(0.13)*			0.899	0.984	-0.01 *(0.13)*			0.944	0.991
Perceived unjust distribution of wealth	-0.23 *(0.05)*			< 0.001	0.791	-0.25 *(0.06)*			< 0.001	0.775	-0.24 *(0.07)*			0.001	0.787	-0.25 *(0.08)*			0.001	0.776
Political interest						-1.25 *(0.08)*				0.288						-0.62 *(0.10)*			< 0.001	0.537

Notes. The engaged class is the reference category. Coeff = Logit coefficient, SE = Standard Error, OR = Odds Ratio

As is shown in Tables 4 and 5 (and Sect.  6 in SOM), ethnic minority/majority group membership differentiated the voter class from the unengaged and the undecided classes: ethnic minority youth were significantly more likely to be in the unengaged and undecided classes than in the voter class (note, however, that in models with parental education, only the latter effect was significant). Ethnic minority/majority group membership did not significantly predict membership in the engaged class (also not in Model 1; but see the “additional analysis” Section below for qualifiers to this). Notably, gender also rather consistently differentiated the classes: Girls were more likely than boys to be in the engaged class and less likely to be in the unengaged class than in all other classes. Regarding socio-economic status indicators, the number of books in the home and attending a gymnasium were rather reliably associated with membership in more politically engaged classes (as did parental education, as reported in Sect.  3 in SOM): both variables predicted a higher likelihood of being in the engaged class over all other classes and a lower likelihood of being in the unengaged class over all or some classes. Youth from more affluent families were significantly more likely to be in the engaged class than the unengaged class, but this effect was no longer significant once political interest was accounted for. Lastly, lower language skills reliably predicted membership in the undecided class over all other classes (but did not differentiate between the other three classes). As expected, youth with higher political interest were more likely to be in the engaged class, and less likely to be in the unengaged class than other classes. Interestingly however, those in the voter and undecided classes did not differ in political interest (with levels of political participation falling in between the engaged and the undecided, see Sect.  4 in SOM for means and standard deviations of predictor variables by class).

Turning to our findings regarding perceived inequality and unfairness, results showed, as expected, that both (i) identifying as a member of a group that is disadvantaged and/or discriminated against and (ii) perceiving more unfair distribution of wealth significantly predicted membership in the engaged class over all other classes (with the exception that - once political interest was accounted for - identification with a disadvantaged and/or discriminated group only marginally differentiated the engaged and voter classes). Identification with a disadvantaged and/or discriminated group further significantly predicted membership in the voter class over the unengaged class (but as Table 4 shows, this effect did not hold in models including political interest).

#### Additional Analyses

To check the robustness of the results, several additional analyses were conducted. First, separate LCAs in subsamples of ethnic minority youth were estimated, to assess whether the four-class solution could be replicated. These LCAs were conducted as an additional robustness check, as some research suggests that ethnic minority and majority youth may be more likely to differ in their *patterns* of political participation than their levels thereof (i.e., what kind of actions they engage in e.g., Stepick et al., 2008), which implies that different classes could plausibly be identified. This analysis is only summarized here (for full model results see Sect.  7.1 in SOM). For the sake of comprehensiveness, ethnic minority group membership was defined in two ways for this descriptive analyses. Firstly, ethnic minority status was defined as having at least one parent born outside Germany (*N* = 894). The four-class solution reported above replicated in this subsample, but the relative sizes of the “unengaged” class was larger and the “engaged” class smaller (about 32% and 14% respectively). Next, only those youth whose both parents were born abroad (*N* = 498) were defined as ethnic minority youth (as in the main analyses). In this case, a three-class solution fit the data well, as separate “voting” and “engaged” classes did not emerge, but rather, a single “open to engagement” class (38%). The other two classes were “unengaged” (52%) and “undecided” (10%). This might reflect that individuals with two foreign-born parents are not automatically granted German citizenship, which is a precondition for eligibility in national elections as adults. Being unsure about whether they will be eligible to vote in the future may thus be one reason explaining why a “voting” class does not emerge among minority students with no German parent.

Second, and relatedly, it was explored whether having German citizenship (or not) differentiated the identified classes in the full sample. This was captured with an available indicator in the data where youth indicated whether they had a German passport (“yes”, “no” or “I don’t know”; 20% indicated they did not know, which were treated as missing in analyses involving this variable). Of the youth that knew the answer, 8% indicated that they did *not* have a German passport. These were almost exclusively ethnic minority youth with two foreign-born parents, of which 30% indicated that they did *not* have a German passport. Subsequent multinomial logistic regression analyses showed that this variable did not significantly predict class membership in model 1. To gauge if this non-finding may have been impacted by our modeling decisions, a simpler model was tested, where ethnic minority (vs. majority) group membership as defined by immigration background was removed from the model, thus defining minority group membership instead exclusively by citizenship. Citizenship did not significantly predict class membership in this model either (see Sect.  7.2 in SOM for further information). These results, together with the substantial missing values, supported the decision not to include this variable in the main analyses.

Lastly, and along similar lines, a simpler version of Model 2 was tested where identification with a disadvantaged/discriminated group and perceived unfair distribution of wealth were not included in the model, to examine whether perceived personal (un)fair treatment significantly predicted class membership in these models. This was not the case (*p*. ≥ 0.365, see Sect.  7.3 in SOM for further information).

## Discussion

Research among adults has clearly shown that examining *patterns* of (envisioned or actual) political participation, that is, how and why different groups of people engage in or prefer certain political actions while avoiding others, can yield valuable insights, advancing understanding of political participation and determinants thereof. Yet, research examining such patterns of envisioned political participation among youth is scarce and often differentiates only between a limited number of potential actions, which may not adequately capture differences in how youth envision their political participation. The current study addresses this gap, examining the envisioned political participation of ethnic minority and majority youth (i.e., with and without a migration background) across secondary schools in Germany. By adopting a person-centered approach and examining numerous forms of political participation, the current work identifies four distinct groups of youth (“classes” in Latent Class Analysis) whose envisioned political participation qualitatively differed. Furthermore, it investigated perceptions of discrimination and unfairness as predictors of envisioned political participation and found that perceptions of systemic and/or group-based discrimination and unfairness reliably predicted membership in more politically engaged classes.

The current work adds to the literature in several ways. Firstly, if envisioned political participation had been examined across the whole sample, only an average pattern of rather high interest in voting and relatively low interest in any other action would have been identified (in the full sample only 14.7% responded “no” to voting, and average “yes” responses for other forms of political participation ranged from 5.8% to 31%). The person-centered approach adopted in the current study yielded a more nuanced picture, distinguishing instead between four groups of youth (latent “classes” in LCA). The largest class of youth showed interest in voting in the future but did not clearly envision engaging in other political actions (“voting” class; 47%). Two further classes consisted of youth that either (i) clearly envi­sioned not only voting but also engaging in varied politi­cal actions in the future (“engaged” class; 20%) or that, in contrast, (ii) did *not* envision engaging in political activities in the future (“unengaged” class, 26%). Lastly, a class of youth who appeared undecided as to whether they would engage in political actions in the future was identified (“undecided” class, 7%). It is worth stating that while youth in the engaged class reported the highest political interest and those in the unengaged the lowest, the undecided youth did *not* have lower political interest than the youth interested in voting, suggesting that other factors would explain their uncertainty. Notably, these classes are conceptually rather similar to four classes identified in a study among young adolescents in the U.S., despite involving a different societal context, time-period, and different indicators of envisioned participation (Wray-Lake et al., 2014). In this U.S. study, the authors further documented that for the majority of youth, these orientations remained stable as they approached adulthood. This suggests that the groups identified in the current study may generalize to further intergroup contexts and may represent already rather stable inclinations to participate politically (but see below for a discussion on changes over time).

Secondly, the currents study adds to the literature by examining how various theoretically relevant variables differentiated between the identified classes (i.e., predicted membership in one class over another), focusing in particular on three kinds of perceptions of discrimination and unfairness. In line with expectations and earlier work (e.g., van Zomeren et al., 2008) perceptions regarding *systemic and/or group based* discrimination and unfairness – that is (i) perceiving that one belongs to a group that faces disadvantage or discrimination and/or (ii) perceiving that wealth is unfairly distributed in society reliably predicted membership in the engaged class over other classes (and over and above variables such as ethnic minority/majority group membership and socio-economic status). In contrast, (iii) more generally perceiving that one is treated (un)fairly in life – in ways that need not be systemic or attributable to one’s social status or group membership – did not predict political participation (in line with e.g., Wood & John-Henderson, 2024). Of course, this should not be taken to mean that unfair treatment or discrimination is in any way desirable, even if it promotes (envisioned) political participation. Instead, *discussing* societal inequalities – i.e., that some groups may face disadvantages and/or negative treatment due to their group membership – can facilitate youth’s awareness of biases and inequalities in society, and thereby promote envisioned political engagement (Schwarzenthal et al., 2022). Including such discussions in school curricula, relating also to unequal distribution of wealth in society, might thus promote political engagement among youth. Importantly, such discussions should simultaneously highlight the benefits of more equal societies that value and respect diversity. Such discussions could promote envisioned political engagement among all youth, while simultaneously promoting the wellbeing and inclusion of ethnic minority youth in diverse schools (Baysu et al., 2025).

The current study findings further showed that socio-economic factors such as educational resources (books in the home) and parental education were associated with a higher likelihood of being in politically engaged classes. Family affluence was less likely to differentiate between the classes, over and above other socio-economic factors. Importantly, while the exploratory LCA in ethnic minority subsamples indicated that ethnic minority youth were less likely to be in the engaged class and more likely to be in the engaged class (a finding that replicated in supplementary multinomial logistic regression models where only ethnic minority/majority group membership and gender were included as predictors of class membership, see Sect.  8 in SOM), the main analysis showed that once such socio-economic factors were accounted for, ethnic minority/majority group membership no longer reliably predicted class membership. In these models, belonging to an ethnic minority group significantly predicted a lower likelihood of being in the voter class over the unengaged and undecided classes, but no longer clearly predicted a lower likelihood of being in the engaged class over other classes, characterized by more global interest in varied future political actions. Thus, these results are in line with earlier work suggesting that (i) failing to account for socio-economic factors and (ii) narrowly defining political participation in terms of voting may underestimate the political engagement of ethnic minority group members (e.g., Riniolo & Ortensi, 2021). Furthermore, ethnic minority youth were more likely to identify as belonging to a group that is disadvantaged or discriminated against, which might promote their envisioned engagement. However, ethnic minority youth perceived – on average – *less* unfair distribution of wealth than their ethnic majority peers. While it is beyond the scope of the current study to speculate why this may be, future research may investigate this further. Another noteworthy finding is that girls were more likely to be in the engaged class than all other classes, followed by the voter class. This contrasts with much research among adults, which often finds that women are less likely to be politically engaged than men, at least when it comes to public and institutionalized activities (Coffé & Bolzendahl, 2010; Zani & Barrett, 2012). This implies that there might be gender-related differences in how intentions to participate translate into actual political participation. Lastly, an interesting finding is that only lower language skills (and not, for instance, lower political interest) reliably distinguished the undecided class from the others. In addition, while ethnic minority/majority group membership did not significantly predict membership in this small class, ethnic minority youth were still overrepresented in this class (see Sect.  4 in SOM). Further research is needed to understand what drives this apparent uncertainty of individuals in the undecided class. It is plausible for instance that factors such as an emerging sense of civic duty among the voting class (Feitosa et al., 2020) and/or uncertainty regarding voting rights may play a role in differentiating these classes.

Alongside contributing to the literature in the above ways, the current work also sets the stage for future research in several ways. Future research may identify classes of (envisioned) political participation and extend our work by investigating what factors may “shift” youth from less engaged classes into more engaged classes, as well as to what extent envisioned engagement translates into actual political participation in adulthood. Prior work has documented that (i) envisioned engagement formed in early adolescence often remains stable as youth approach adulthood (Jungkunz & Marx, 2025; Wray-lake et al., 2014), and that (ii) envisioned engagement meaningfully predicts actual participation later in life (e.g., Quintelier & Blais, 2016). Yet, the extent to which envisioned participation translates into actual participation is likely to vary depending on a number of factors which continue to develop and unfold in (later) adolescence and that future studies may investigate. Such research may consider not only perceived discrimination, unfairness, and awareness of societal inequalities – which the current as well as other research suggests may promote envisioned engagement among youth (e.g., Schwarzenthal et al., 2022) – but also, e.g., adolescents’ (internal and/or external) political efficacy (e.g., Eckstein et al., 2013; Garritzmann et al. 2025), perceived group norms and social networks (e.g., González, 2025) and politicized identities (Árnadóttir et al., 2025; van Zomeren et al., 2008). Relatedly, future research may investigate the role of contexts, including for instance classroom- and societal climates, societal systems and structures (Schwarzenthal et al., 2022; Vráblíková and Císar, 2015), which undoubtedly play a role in informing both (envisioned) political participation and perceptions of discrimination and unfairness, but which fall beyond the scope of the current study. For instance, our findings may be influenced by the fact that, in comparison to many other countries, Germany has a highly stratified education system that rigidly places students into different educational tracks between ages 10 and 12 (Helbig & Nikolai, 2015). This leads, in comparison to other country contexts, to higher political participation gaps between higher and lower social strata (Garritzmann & Wehl, 2025), and a more pronounced disadvantage of ethnic minority students in the education system (Pomianowicz, 2023).

The current study has some limitations, some of which relate to the fact that the data used was not collected with this study in mind. Firstly, many of our constructs were measured with single-item indicators. While not optimal, this is common practice in large-scale school surveys for obvious pragmatic reasons. Moreover, perceived discrimination and unfairness are commonly measured with one single question (e.g., Jetten et al., 2001). Second, while the political participation items in the current study covered a range of actions, those items were limited in that they did not capture the goal of the actions, e.g., *to what ends* youth would participate in a demonstration (see e.g., Osborne et al., 2019). Third, the cross-sectional design limits any causal inferences, regarding, for example, the causal role of perceived discrimination and unfairness in promoting envisioned political participation. While this is certainly a limitation, the findings of the current study align both with socio-psychological theories, and with prior longitudinal evidence (e.g., Plummer et al., 2022). A fourth limitation relates to the operationalization of ethnic minority (vs. majority) group membership, where heterogeneous “ethnic minority” youth from various countries, which differed in whether they were born in Germany for instance, were grouped together. However, the person-centered approach of the current study, which involved detecting subgroups in the full sample, followed by the *exploratory* examination of ethnic minority/majority group membership justifies this. Nevertheless, this operationalization likely obscures considerable heterogeneity in terms of e.g., origins, sense of belonging to Germany, socio-economic status, and intersections thereof, which can inform political participation. Indeed, a large project investigating the political participation of young people across several European countries reported that while ethnic minority group members were sometimes less politically engaged than ethnic majority peers, this was by no means always the case, as there was considerable variation depending on factors such as for instance gender, country of origin, and intersections thereof (Barrett, 2012). While examining such intersections falls beyond the scope of the current work, researchers should engage critically with definitions of (ethnic) group memberships to avoid perpetuating oversimplified views on group differences, and to allow for the detection of meaningful differences *within* such categories (see Civitillo et al., 2025).

## Conclusion

Research among adults has clearly illustrated the value of examining *patterns* of (envisioned or actual) political participation, that is, how and why different groups of people engage in or prefer certain political actions while avoiding others, but such research remains more limited among youth. To address this gap, the current study investigated how youth envision their future political engagement at an early stage of adolescence, utilizing a large school-based sample in Germany. By adopting a person-centered approach and differentiating between varied forms of political participation, four latent classes of youth that differed in their envisioned political engagement were identified: “voting” (47%), “unengaged” (26%), broadly “engaged” (20%), and “undecided” (7%). Perceptions of systematic and/or group-based discrimination and unfairness, that is, perceiving (i) that one belongs to a group that is disadvantaged and/or discriminated against in society and (ii) that wealth is unfairly distributed in society robustly predicted membership in the engaged class over other classes. This suggests that such perceptions can promote intentions to engage politically among youth, potentially mobilizing them as “future activists”. While this should not be taken to mean that societal inequalities or experiences of discrimination are desirable as motivators of political participation, the current study findings align with earlier work suggesting that promoting an *awareness* of societal inequalities (e.g., through school discussions) can promote envisioned engagement among youth. Schools and educators should thus be encouraged to include discussions of systemic inequalities and unfairness in school curricula, while simultaneously highlighting the benefits of more equal societies that value and respect diversity.

## Declarations

### Data Sharing Declaration

The data and syntax used for the current study will be made available via OSF upon acceptance of the article for publication.

### AI Declaration

No generative AI was used in the development of this article.
